# African immigrants with type 2 diabetes present with three physiologic subtypes: implications for screening, diagnosis and treatment

**DOI:** 10.1136/bmjdrc-2025-005504

**Published:** 2026-03-18

**Authors:** Eliza A Huefner, Kauthrah Ntabadde, Grace G Smith, Simon Pierre Bigirimana, Christopher W DuBose, Arthur Sherman, Anne E Sumner

**Affiliations:** 1 Section on Diabetes, Nutrition, and Health in the Diabetes, National Institute of Diabetes and Digestive and Kidney Diseases, Bethesda, Maryland, USA; 2 National Institute on Minority Health and Health Disparities, Bethesda, Maryland, USA; 3 LBM, NIDDK, NIH, Bethesda, Maryland, USA; 4 Hypertension in Africa Research Team, North-West University, Potchefstroom, North-West, South Africa

**Keywords:** Insulin Resistance, Insulin Secretion, Glucose Tolerance Test

## Abstract

**Introduction:**

As type 2 diabetes (T2D) prevalence increases in the USA and Africa, factors from both regions affect African immigrants.

**Objective:**

T2D in African immigrants was characterized by examining: (a) insulin deficiency and insulin resistance; (b) phenotypic presentation; (c) sociodemographic factors.

**Methods:**

In 633 African immigrants (male: 62%, age 39±11, (mean±SD), range 20–70 years), body mass index (BMI): 27.8±4.6, range 18.8–46.2 kg/m^2^), weight gain information was collected, BMI and waist circumference (WC) measured and OGTT performed. Insulin resistance was defined by the lowest quartile of the Matsuda Index (≤2.80); insulin secretion by the Insulin Secretion Index (ISI). Insulin deficiency was defined as less than the maximum ISI in participants with T2D without IR (0.430). WC thresholds defined central obesity (men: WC ≥94 cm; women ≥80 cm).

**Results:**

Normal glucose tolerance, pre-diabetes and T2D occurred in 61%, 32% and 7%, respectively. Three subtypes of T2D were identified: insulin-deficient-T2D (ID-T2D) in 45%, insulin-resistant-T2D (IR-T2D) in 30%, insulin-deficient+insulin-resistant (ID+IR-T2D) in 25%. ID+IR-T2D had the highest glucose concentrations (all p<0.05), whereas insulins were highest in IR-T2D (all p<0.01). Phenotypic differences by T2D subtype were identified. In the ID-T2D group, 20% of participants had a healthy weight and central obesity occurred in 55%. In the IR-T2D and ID+IR-T2D groups, 100% had central obesity and a BMI in either the overweight or obese categories. Sociodemographic factors specifically, weight gain, sedentary lifestyle and percent married, increased across glucose tolerance category (p values <0.01) but did not differ by T2D subtype (p≥0.3).

**Conclusions:**

Spanning the BMI spectrum from normal to obese, African immigrants have three subtypes of T2D. Weight gain was greatest in immigrants who developed T2D but did not differ by subtype. As life in America promotes weight gain, sharing information about the consequences of weight gain with all Americans, both native and foreign-born, is key to T2D prevention and treatment.

**WHAT IS ALREADY KNOWN ON THIS TOPIC**

- The prevalence of type 2 diabetes (T2D) is rapidly rising in both Africa and the United States. Therefore, the growing population of African immigrants to the United States is exposed to risk factors for T2D from both regions. As only minimal information on T2D in African immigrants is available, the design of effective prevention and treatment requires investigations, which consider the specific and unique cultural, sociodemographic and metabolic risk factors they face.

**WHAT THIS STUDY ADDS**

- Metabolically, we found in African immigrants there are three distinct physiologic subtypes of T2D: (1) insulin-deficient, (2) insulin-resistant and (3) insulin-deficient and insulin-resistant combined. The most common T2D subtype was insulin-deficient, and it frequently occurred in African immigrants who were healthy weight and did not have central obesity. In addition, across T2D subtypes, we identified three factors related to life in the United States, which were markedly higher in immigrants who had T2D compared with those who did not: weight gain, decreased physical activity and a higher marriage rate.

**HOW THIS STUDY MIGHT AFFECT RESEARCH, PRACTICE OR POLICY**

- Screening for T2D in African immigrants should be focused across the body mass index (BMI) spectrum, from healthy weight to obesity, and not only on individuals with a BMI ≥25 kg/m^2^ as is current practice in the United States. In addition, as two of the T2D subtypes are insulin-deficient, insulin therapy may be needed early in the course of treatment of African immigrants with T2D. Furthermore, as marriage increased in frequency across the glucose tolerance categories, engaging family units rather than individuals in programs about diet and exercise could be very beneficial.

## Introduction

 Between 2024 and 2050, the International Diabetes Federation (IDF) projects that the number of individuals in sub-Saharan African countries with type 2 diabetes (T2D) will increase from 24.6 million to 59.5 million.^1^ This 142% increase in T2D is the highest anticipated increase in the world.^1^ In addition, the IDF estimates that 73% of individuals living with T2D in Africa are undiagnosed.^1^ Reasons for undiagnosed T2D include limited access to care and the poor diagnostic performance in African descent populations of both hemoglobin A1C (HbA1c) and fasting plasma glucose (FPG).^2^,^6^ With undiagnosed T2D common in Africa, it is anticipated that some Africans who immigrate to the USA will have undiagnosed T2D. In addition, new cases of T2D will develop after immigration because life in the USA is characterized by social, financial and cultural stress from the transition from a majority to minority population as well as a sedentary lifestyle, increased exposure to calorie-dense processed food and weight gain.^7^

As T2D complications are preventable but not reversible, early detection of T2D is imperative.^1 8^ Currently, information on T2D in African immigrants is scant. While T2D studies have been conducted in Africans living in the USA and Europe, they rely on FPG or self-report of T2D, and therefore, subjected to under-reporting.^9^,^11^

Studies in African Americans cannot offset the need for African immigrant data.^12^ Similarly, while studies conducted in African countries have brought insight, they cannot assess the influence of the American diabetogenic environment.^13^,^16^

To address the T2D data gap in African immigrants, we turned to the oral glucose tolerance test (OGTT). For T2D diagnosis, the OGTT is highly reproducible and when conducted as a multisampled test, both insulin deficiency and insulin resistance can be identified.^17 18^

We performed a multisampled OGTT in Africans in America Study enrollees and T2D was characterized by examining the: (a) balance between insulin deficiency and insulin resistance; (b) phenotype and (c) sociodemographic determinants.

## Methods

As described previously, the Africans in America Study were designed to identify determinants of T2D in African-born blacks living in the USA.^219^,^22^ NIH Institutional Review Board approved the protocol (Clinical Trials.gov Identifier: NCT00001853). Enrollees gave informed consent.

Enrollees had to be born in Africa, self-identified as black, aged 18–70 years, currently live in metropolitan Washington, DC and report both parents were born in Africa. Persons previously diagnosed with T2D were not enrolled.

At visit 1, history and physicals were performed. Blood tests were done including hemoglobin, creatinine, liver function tests and thyroid-stimulating hormone (TSH). Questions about sociodemographic data were asked of every participant in a standardized uniform fashion in the same order and included immigration age, years in USA, weight gain in USA, physical activity, marital status (meaning currently married vs single, separated, divorced or widowed), alcohol intake, current smoker (yes/no), income (>45 k) and college graduate (yes/no). Exercise was assessed with the International Physical Activity Questionnaire (IPAQ) and dichotomized as sedentary (IPAQ category: low) or active (IPAQ categories: moderate or high).^23^

Visit 2 took place within 2 weeks of visit 1. Weight, height, BP, waist circumference (WC) and HbA1c were obtained. OGTT (Trutol75, Custom Laboratories) was performed with glucose and insulin obtained at baseline, 30, 60 and 120 min. C-peptide levels were available in 496 of 633 participants. Anti-insulin and GAD65 antibodies were obtained in the last eight persons with T2D.

### Determination of glucose tolerance

T2D diagnosis required FPG ≥126 mg/dL and/or 2-hour glucose ≥200 mg/dL; pre-diabetes: FPG ≥100–125 mg/dL and/or 2-hour glucose ≥140 mg/dL and <200 mg/dL; normal glucose tolerance (NGT): FPG <100 mg/dL and 2-hour glucose <140 mg/dL.^8^

### Body size and visceral adiposity

BMI categories were underweight: BMI<18.5 kg/m^2^; healthy weight: BMI 18.5–24.9 kg/m^2^; overweight: BMI 25.0–29.9 kg/m^2^; obesity: BMI≥30 kg/m^2^.^24^

Both WC and visceral adipose tissue (VAT) were assessed. WC was measured standing at end-expiration at the superior border of the iliac crest.^25^ Central obesity sex-specific thresholds were for men: WC ≥94 cm and women: WC ≥80 cm).^26^

To measure VAT, computerized tomographic scans (Siemens and Somatom Force Scanner) were performed at L2–3 with automated software in 555 consecutively enrolled participants.^27^

### Insulin resistance

Diagnostically, insulin resistance was defined as the value below the lowest quartile of the population distribution for the Matsuda Insulin Sensitivity Index (≤2.80).^28^

### Insulin secretion and insulin deficiency

Insulin secretion was assessed by the insulin secretion index (ISI).^2^

The insulin-deficiency threshold was defined as the highest value of the ISI (<0.43) at which T2D occurs in the absence of insulin resistance (Matsuda Index >2.80).

### Disposition index

Disposition Index (DI), a measure of the ability of the beta cell to overcome insulin resistance, was the product of ISI times Matsuda Index.^29^

### Assays

Hemoglobin was measured in EDTA-anticoagulated whole blood (Sysmex XE-5000). Alanine and aspartate aminotransferases were measured in plasma using Nicotinamide Adenine Dinucleotide Hydrogen (NADH) in the presence of pyridoxal-5-phosphate (Abbott Architect). Glucose was measured in plasma (Abbott Architect). TSH was measured with a two-step Chemiluminescent Microparticle Immunoassay (Abbott Architect).

Insulin and C-peptide were measured in serum using a Roche Cobas 6000 analyzer (Roche Diagnostics). HbA1c values were determined by HPLC using NGSP-certified instruments (BioRad Laboratories). GAD65 and anti-insulin antibodies were measured by radioimmunoassay (Perkin Elmer Gamma Counter Wizard 2).

### Statistical analyses

Unless stated otherwise, data presented as mean±SD. Continuous variable comparisons were by one-way analysis of variance (ANOVA) with Bonferroni corrections for multiple comparisons. Categorical variable comparisons were by χ^2^. The Jonckheere–Terpstra test was used to determine the p value for trend across glucose tolerance categories.

To examine the influence of sociodemographic characteristics and health behaviors, three multivariate logistic regression models were fitted, with T2D as the outcome. Statistical significance was assessed using Wald test p values.

Model 1 included the entire cohort (n=633) and evaluated sociodemographic factors: age (continuous), marital status (married vs not married), education level (college degree vs no college degree) and annual household income (≥45 k vs <45 k).

Model 2 included the consecutively enrolled participants with IPAQ data (n=426). This model adjusted for all variables in model 1 with addition of physical activity level (sedentary vs active).

Model 3 included weight gain in addition to all variables in model 1 and was restricted to participants who migrated to the USA ≥18 years.

P values ≤0.05 were considered significant. Data were managed with Research Electronic Data Capture (REDCap).^30^ Analyses were performed with STATA V.19.0.

## Results

Seven hundred immigrants attended visit 1. Eight per cent, mostly related to anemia, pregnancy and scheduling conflicts did not proceed to visit 2 (online supplemental Figure 1). Hypothyroidism, a metabolic reason for weight gain, led to one person with an elevated TSH to be excluded. Of the remaining 645 enrollees, 2% had missing glucose or insulin samples. The final cohort had 633 participants (male: 62%, age: 39±11, range 20–70 years, BMI: 27.8±4.6 (mean±SD), range 18.8–46.2 kg/m^2^). Participants’ African region of origin was West: 49%, East: 32%, Central: 17% or South: 2% (online supplemental Figure 2).

### Glucose tolerance status

NGT, pre-diabetes and T2D were diagnosed in 61%, 32% and 7% of participants, respectively (online supplemental Table 1)

### Studies to detect type 1 diabetes

Anti-insulin and GAD65 antibodies were undetectable in the eight *participants* with T2D and antibodies available.

Forty *participants* had both T2D and C-peptide levels measured. Postglucose load C-peptide concentrations were >600 pmol/L.^8^

### Physiology of T2D

**Figure 1:**
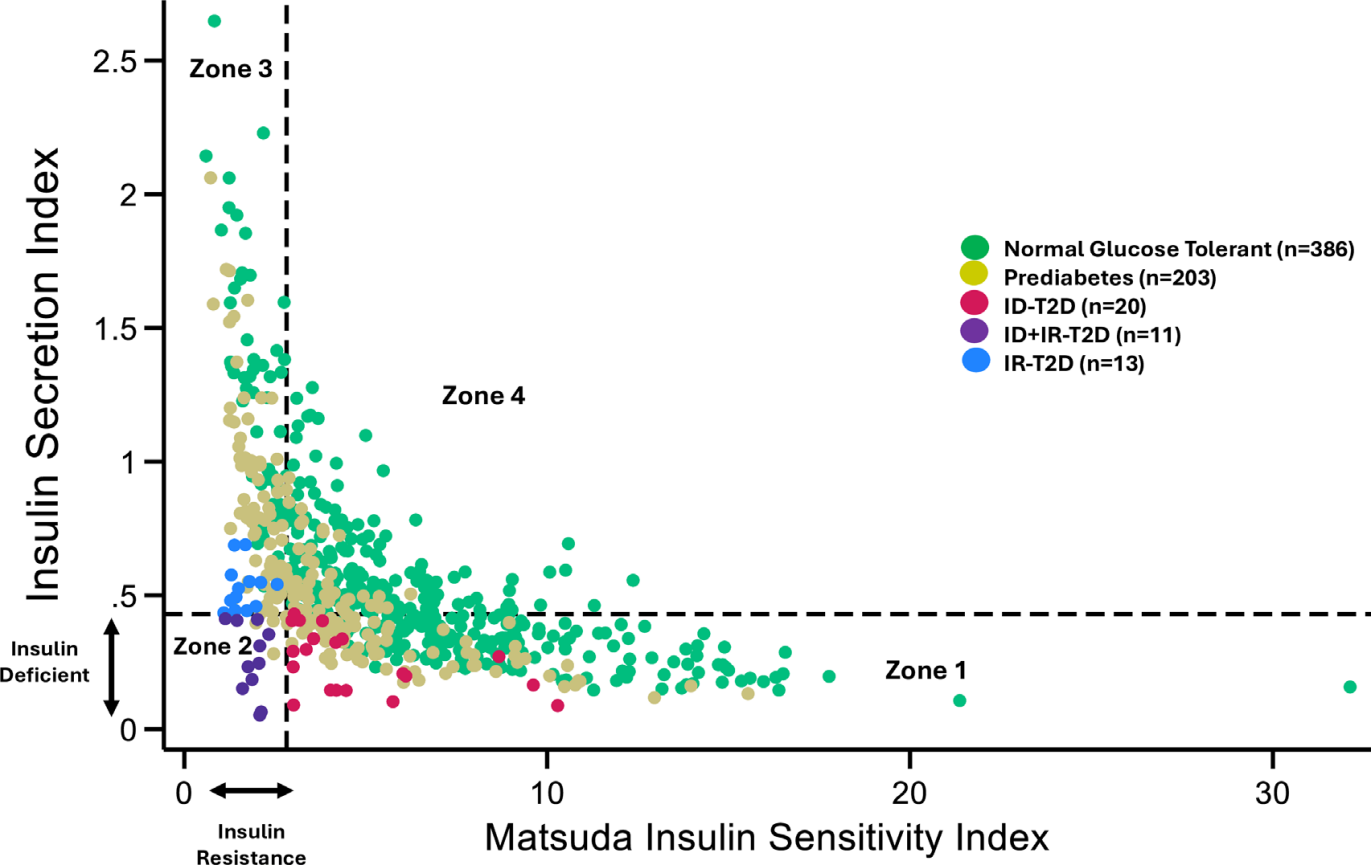
Disposition Index: Insulin Secretion Index vs Matsuda Index. Glucose tolerance categories of participants are illustrated by color dots: Green: normal glucose tolerance; khaki: pre-diabetes; red: ID-T2D; purple: ID+IR-T2D; blue: IR-T2D. Threshold for insulin deficiency is designated by horizontal dashed line at 0.43. Threshold for insulin resistance is demarcated by dashed vertical line at 2.80. Zone 1: participants without insulin resistance but with insulin concentrations in the range that was too low to prevent T2D in 20 individuals. zone 2: participants with insulin resistance and insulin deficiency. zone 3: participants with insulin resistance and zone 4: participants without insulin resistance or insulin deficiency. ID, insulin-deficient; IR, insulin-resistant; T2D, type 2 diabetes.

Three T2D subtypes were identified after the graph of DI *for the entire cohort* was divided into four zones according to population-specific thresholds for insulin secretion (ISI<0.43) and insulin resistance (Matsuda ≤2.80) (figure 1). The subtypes were insulin-deficient-T2D (ID-T2D): 45% (20/44), insulin-resistant-T2D (IR-T2D): 30% (13/44), insulin-deficient+insulin-resistant-T2D (ID+IR-T2D): 25% (11/44).

Zone 1 includes participants (n=264) with insulin deficiency but not insulin resistance. Twenty of the 264 participants in this zone had T2D because of insulin deficiency—meaning their insulin concentrations were too low to prevent T2D in the absence of insulin resistance. Zone 2 includes the participants (n=13) with both insulin deficiency and insulin resistance and most (n=11) had T2D. Zone 3 is characterized by insulin resistance alone (n=143) and includes the participants with T2D from insulin resistance (n=13). Zone 4 includes the participants without insulin deficiency or insulin resistance (n=213). No participants in zone 4 had T2D.

**Figure 2:**
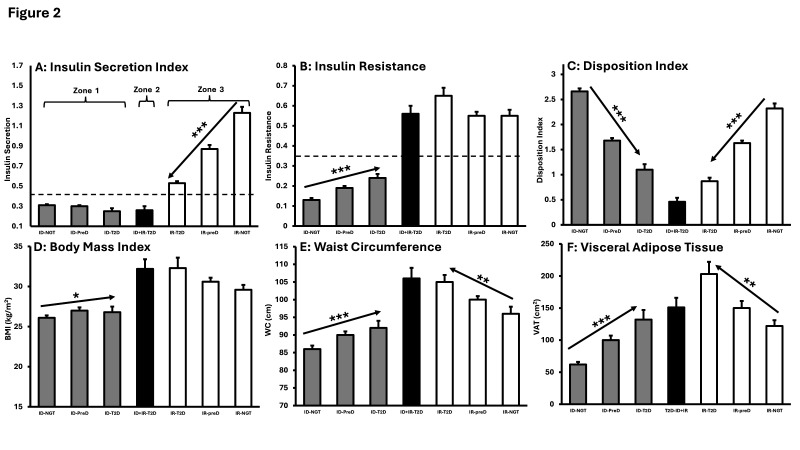
Characteristics by glucose tolerance category and T2D subtype. Gray columns: insulin deficient groups; black columns: DM due to ID+IR, white columns: insulin resistant groups (**A**) Insulin Secretion Index: horizontal dashed line at 0.43 representing the threshold below which criteria for insulin deficiency was met; (**B**) insulin resistance presented as 1/Matsuda Index with horizontal dashed line at 0.35 representing the threshold above which insulin resistance occurs; (**C**) Disposition Index; (**D**) body mass index (BMI); (**E**) waist circumference (WC); (**F**) visceral adipose tissue (VAT). Arrows illustrate significant trends across glucose tolerance categories from NGT to pre-diabetes to T2D. *p≤0.05, **p≤0.01, ***p≤0.001. Data presented as mean±SE. ID, insulin-deficient; IR, insulin-resistant; NGT, normal glucose tolerance; T2D, type 2 diabetes.

Differences in insulin deficiency, insulin resistance and DI were detected within zones 1, 2 and 3. In zone 1, insulin secretion did not change across glucose tolerance categories (figure 2A, first three columns; online supplemental Table 2), but insulin resistance increased (figure 2B: first three columns). In zone 3, insulin resistance did not change across glucose tolerance categories (figure 2B, last three columns) and insulin secretion declined (figure 2A, last three columns).

DI was lowest in the participants in zone 2 with ID+IR-T2D (figure 2C, middle column). However, DI declined significantly across glucose tolerance categories in zone 1 and zone 3 (figure 2C, both p values for trend <0.001). DI did not differ between ID-T2D (zone 1) and IR-T2D (zone 3) (p=0.288).

BMI increased across glucose tolerance categories in zone 1 (figure 2D and p value for trend=0.011) but remained below the levels observed in zones 2 and 3. WC and VAT increased across glucose tolerance categories in both zones 1 and 3 (figure 2E–2F) (all p values for trend <0.01).

### Glucose and insulin concentration differences by T2D subtype

**Figure 3:**
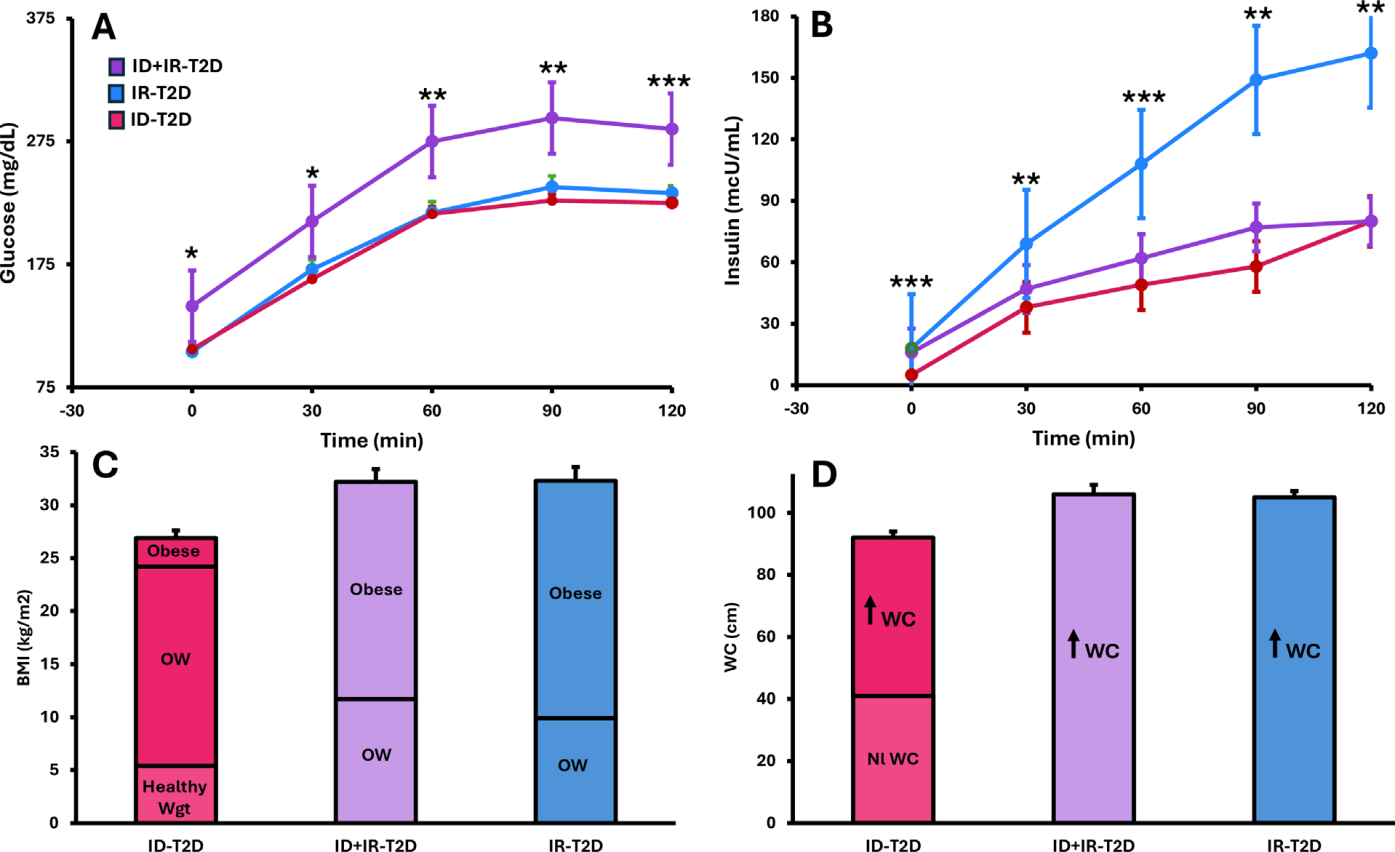
Physiologic and phenotypic characteristics by T2D subtype. Red: data from participants with ID-T2D, purple: data from participants with ID+IR-T2D, blue: data from participants with IR-T2D. (**A**) Glucose concentrations during OGTT; (**B**) insulin concentrations during OGTT; (**C**) BMI category: healthy weight (Nl wgt), overweight (OW), obesity; (**D**) waist circumference (WC): normal WC (Nl WC), elevated WC (↑ WC). WC thresholds adjusted for gender. *p≤0.05, **p≤0.01, ***p≤0.001. Data presented as mean±SE. BMI, body mass index; ID, insulin-deficient; IR, insulin-resistant; OGTT, oral glucose tolerance test; T2D, type 2 diabetes.

Throughout the OGTT, glucose concentrations were highest in the ID+IR-T2D subtype and did not differ between the ID-T2D and IR-T2D subtypes (all p>0.9) (figure 3A).

Insulin concentrations were highest in the group with IR-T2D (figure 3B) and did not differ in the ID-T2D and ID+IR-T2D subtypes (all p>0.3).

### Variations in body size and fat distribution by T2D subtype

In the ID-T2D group, 20% had a healthy weight, 70% had overweight and 10% had obesity (figure 3C). In the ID+IR-T2D group, 36% had overweight and 64% had obesity. In the IR-T2D group, 31% had overweight and 69% had obesity.

In the ID-T2D group, 55% of participants had central obesity. In the ID+IR-T2D and IR-T2D groups, 100% had central obesity (figure 3D).

#### Sociodemographic and health behaviors

Sociodemographic and health behaviors were examined in two phases. The first phase included all participants (n=633). The second phase was restricted to participants with T2D (n=44).

**Figure 4:**
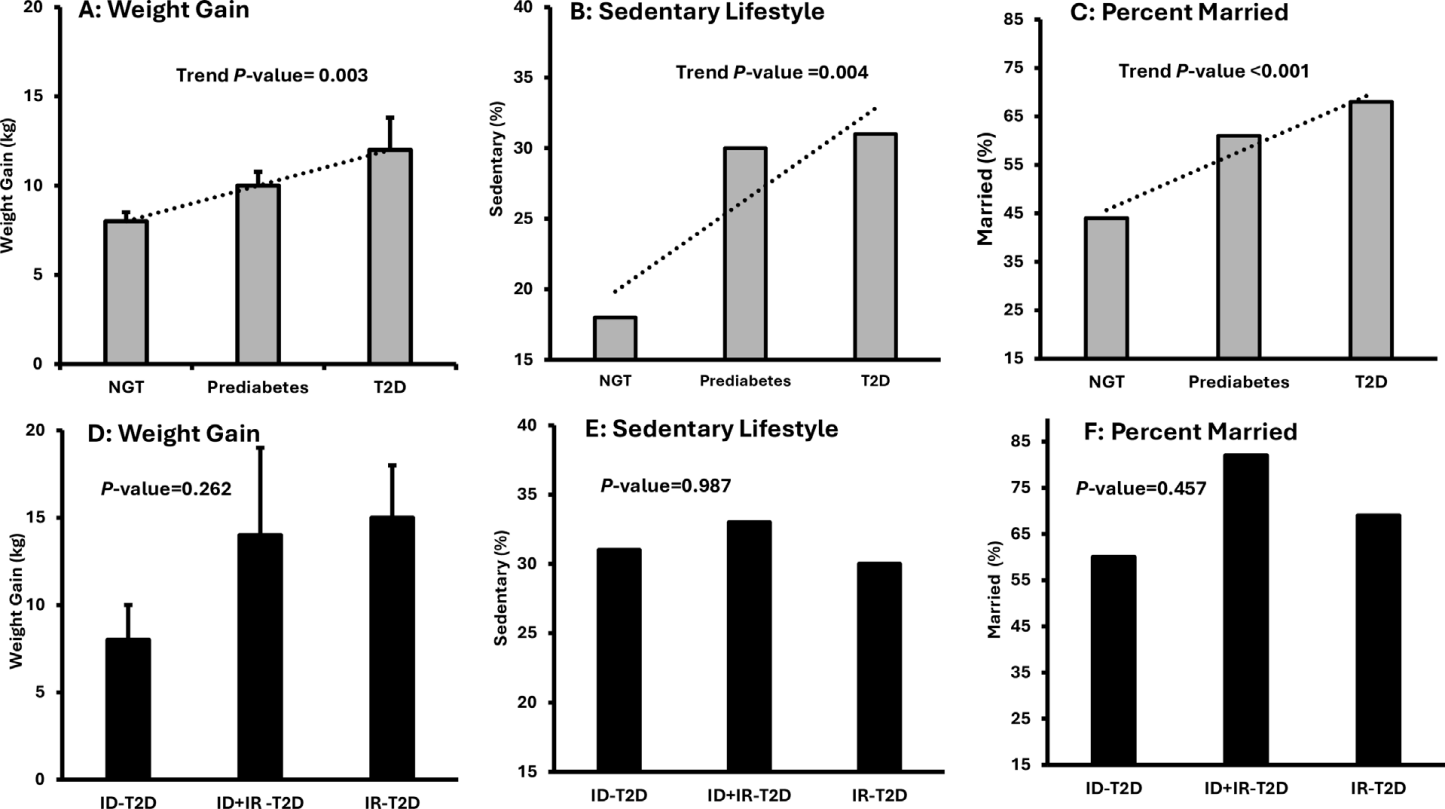
Participant demographics. (A–C) Glucose tolerance categories. (D and E) Diabetes subtype. Data for continuous variables presented as mean±SE. P values for (A, B and C) are for trend across glucose tolerance categories. P values for (D, E and F) are for one-way ANOVA between T2D subtypes. ANOVA, analysis of variance; ID, insulin-deficient; IR, insulin-resistant; T2D, type 2 diabetes.

In phase 1 analyses, NGT, pre-diabetes and T2D were examined as a series and there was a significant upward trend in age, immigration age, years in USA, weight gain, sedentary lifestyle and per cent married (figure 4A–C, online supplemental file 1). Alcohol intake tended to increase across glucose tolerance categories (p=0.057), while smoking, income and education did not (all p≥0.3) (online supplemental Table 1).

In all three of the multivariable logistic regression models, age was independently associated with T2D, but education and income were not (online supplemental Table 3).

In the subset with IPAQ data (model 2), sedentary lifestyle was not associated with T2D (aOR 1.24, 95% CI 0.54 to 2.84, p=0.612).

In model 3, which was restricted to participants who immigrated ≥18 years, weight gain was independently associated with higher odds of T2D (aOR 1.03, 95% CI 1.00 to 1.06, p=0.048).

In all three models, marital status showed a non-significant trend towards higher odds of T2D (model 1: aOR 1.74, 95% CI 0.87 to 3.48, p=0.121; model 2: aOR 1.85, 95% CI 0.79 to 4.36, p=0.158; model 3: aOR 2.04, 95% CI 0.94 to 4.44, p=0.074).

Phase 2 analyses focused exclusively on the T2D group. By 1-way ANOVA, there were no significant differences by T2D subtype in any sociodemographic or health behavior factors including age, weight gain, sedentary, per cent married, education or income (table 1, figure 4).

**Table 1:** Characteristics of participants with diabetes

Parameter	Diabetes n=44 100%	ID-T2D n=20 45%	ID+IR-T2D n=11 25%	IR-T2D n=13 30%	P value†
Basic demographics and metabolic characteristics					
Male (%)	75%	85%	64%	69%	0.358
Age (years)	45±10	45±10	44±10	45±10	0.991
BMI (kg/m^2^*)	29.8±4.5	26.8±2.9	32.2±3.9	32.3±4.6	<0.001 a***b***
Obesity (%)	41%	10%	64%	69%	0.001
Healthy weight (%)	9%	20%	0%	0%	0.003
WC (cm)	99±10	92±7	106±9	105±8	<0.001 a***b***
Central obesity (%)	80%	55%	100%	100%	0.001
VAT (cm*) (n=37)	158±64	132±60	151±46	203±65	<0.05b**
Matsuda index (inverse)	0.44±0.22	0.24±0.08	0.56±0.13	0.65±0.15	<0.001 a***b***
Insulin Secretion Index	0.34±0.17	0.25±0.11	0.26±0.13	0.53±0.09	<0.001b***c***
Disposition Index	0.87±0.44	1.10±0.47	0.46±0.25	0.87±0.26	<0.001 a***c*
Sociodemographic and health factors					
Income (>45K) (%)	59%	45%	73%	69%	0.219
College graduate (%)	68%	65%	73%	69%	0.903
Married (%)	68%	60%	82%	69%	0.457
Age of immigration (years)*	31±10	29+9	32+12	33±9	0.525
Years in USA (years)*	15±11	18±10	13+13	13±9	0.357
Weight gain in USA (kg)*	12±12	8±8	14±17	15±10	0.262
Sedentary (%) (n=32)	31%	31%	33%	30%	0.987
Alcohol intake≥7drk/wk	6%	20% (4/20)	0% (0/11)	15% (2/13)	0.293
Smoker (%)	14%	25%	0%	8%	0.115

One-way ANOVA for continuous variables, *p≤0.05. **p≤0.01, ***p≤0.001. a for difference between ID-T2D and ID+IR-T2D, b for difference between ID-T2D and IR-T2D, c for difference between ID+IR-T2D and IR-T2D.

* Came to the USA as adult ≥18 years (n=40).

† χ2 for categorical variables.

ANOVA, analysis of variance; BMI, body mass index; ID, insulin-deficient; IR, insulin-resistant; T2D, type 2 diabetes; VAT, visceral adipose tissue; WC, waist circumference.

## Discussion

As the first investigation in African immigrants to use multisampled-OGTT to study glucose tolerance physiology, three T2D subtypes were identified as well as three factors, which could be leveraged for T2D treatment and prevention. An awareness that T2D subtypes occur and that they differ in both phenotype and physiology, could lead to better screening paradigms and earlier institution of optimal medical therapy. The three factors that could be leveraged, independent of subtype, to treat or prevent T2D, were weight gain, sedentary behavior and marriage, which we view as a proxy for the opportunity to work with family units.

The three T2D subtypes identified were ID, IR and ID+IR. Screening practice is affected. The ADA recommends screening for T2D for individuals with a BMI ≥25 kg/m^2^.^8^ However, ID-T2D was the most common subtype, and BMI in these participants was often <25 kg/m^2^. Diagnosis of T2D in African immigrants should not be delayed due to a lack of appreciation of the T2D risk even when BMI <25 kg/m^2^. T2D in individuals with a BMI <25 kg/m^2^ is also characteristic of populations of Asian descent.^31 32^

Recognition of T2D subtype can also contribute to early optimization of hypoglycemic medication. Insulin concentrations were lowest in the ID-T2D and ID+IR-T2D. Both insulin-deficient groups are likely to need insulin therapy even though the latter often have a BMI in the obese category, and the former not. While oral hypoglycemics are typical initial therapy for newly diagnosed T2D individuals with obesity, the subgroup with ID+IR-T2D may need early transition to insulin. Practitioners need to be aware that this need for insulin in some individuals with T2D and obesity is not due to patient non-compliance with oral therapy, but to the physiologic presence of insulin deficiency.

### Factors that can be modified or leveraged for treatment and prevention

Age, age of immigration, years in USA, weight gain, sedentary lifestyle and marriage, all trended significantly upward across glucose tolerance categories (online supplemental Table 1). However, by T2D subtype, there were no differences in any of these factors (all p>0.3). Two of these risk factors are modifiable, weight gain and sedentary lifestyle, and marriage represents an opportunity (table 1).

Weight gain is a potent global risk factor for developing T2D.^1^ As the USA is an obesogenic environment, African immigrants across the BMI spectrum should prospectively receive public health information about T2D risk from weight gain.^7^ Second was physical activity. Life in America is sedentary.^7^ While we found that low physical activity did not increase the odds of T2D, increasing physical activity is the bedrock of both treatment and remission strategies.^33 34^ As increasing physical activity promotes weight loss, decreases insulin resistance and improves degree of glycemia, African immigrants, and indeed, all Americans, should have the benefit of health education about diet and exercise.^33^

The third factor was marriage. Marriage does not promote T2D. However, in some African societies, marriage may be associated with weight gain, and this is often attributed to a preference for large body sizes.^35^,^38^ Overweight may be seen in women as a sign of fertility and in men, both prosperity and contentment.^35^,^38^ The high marriage rate in African immigrants represents an opportunity for teaching the whole family about nutrition, healthy weight and exercise.

### Physiology of T2D subtypes

We were able to identify the three T2D subtypes because of our ability to graph zones for DI (figure 1). Validity of these zones is demonstrated by the plot of insulin secretion versus insulin resistance as: (a) the expected hyperbolic relationship occurred and (b) across glucose tolerance categories, there was a distinct leftward and downward shift. All participants with T2D were in zones 1, 2 or 3.

The most frequent T2D subtype was ID-T2D, occurring in 45%, and localized to zone 1. In zone 1, ISI was low and did not change across glucose tolerance categories, but IR increased (figure 2A–B, online supplemental Table 2). As BMI, WC and VAT increased across glucose tolerance categories, weight gain is the probable etiology for this increase in insulin resistance (figure 2D–E). Nonetheless, 20% of the participants in this subtype were healthy weight and only 55% were centrally obese.

Insulin deficiency in Africans most likely reflects decreased insulin secretion per beta cell and decreased beta cell mass. The most likely causes are early life undernutrition, environmental toxins, infectious diseases and genetic factors.^12 13^

The second most common T2D subtype, comprising 30%, occurred in zone 3. This subtype is characterized by hyperinsulinemia and insulin resistance. Within zone 3, insulin resistance did not change, but insulin secretion declined (figure 2A–B). This decline in insulin secretion leads to hyperglycemia because of decreased ability of beta cells to compensate for insulin resistance.^39^ Nonetheless, insulin concentrations in the IR-T2D group were significantly higher than in the ID-T2D or ID+IR-T2D subtypes (figure 3B).

Phenotypically, all participants with IR-T2D were either overweight or obese (figure 3C), and 100% had central obesity (figure 3D). The high rate of central obesity is directly related to significant increases across glucose tolerance categories in both WC and VAT (figure 2E–F).

The third subtype of T2D was ID+IR-T2D, in zone 2, and occurred in 25% of participants with T2D. This subtype represents a combination of insulin deficiency and insulin resistance. In this subtype, glucose concentrations were highest and DI lowest.

As ID+IR-T2D was characterized by both insulin deficiency and insulin resistance, we believe that transition from either ID-T2D or IR-T2D to ID+IR-T2D is possible. While we are not aware of any prospective studies on diabetic physiology in Africans, studies in other populations have shown that with longstanding insulin resistance, and independent of the cause of insulin resistance, β-cell exhaustion occurs, and the ultimate result is a transition from IR-T2D to ID+IR-T2D.^39^,^44^ Therefore, African immigrants with IR-T2D are likely to transition to ID+IR-T2D.

In contrast, to studies of IR-T2D, no prospective studies in people of African descent of ID-T2D are available. However, with weight gain, and the concomitant development of insulin resistance, participants with ID-T2D may transition to ID+IR-T2D (zone 1 to zone 2). As African-born blacks who move to either the USA or Europe have marked increases in weight, they may be at especially high risk for this transition.^7 45^ The increases in BMI, WC and VAT we observed across glucose tolerance categories in the ID-T2D subtype support the hypothesis that transitions from ID-T2D to ID+IR-T2D are likely.

### Comparative studies

Our work determining physiologic T2D subtypes in African immigrants expands and reinforces observations made in African countries, African immigrants to Europe, and diabetes clustering analyses performed in Asian Indians and Swedes.^13 15 16 31 46 47^

The three T2D subtypes identified in African immigrants have also been observed to varying degrees in Tanzanian, Ugandans and South African. PrayGod *et al* performed OGTT in 1890 Tanzanians with a history of TB or HIV and enrollment in a nutritional supplementation trial.^16^ Seven per cent of participants had T2D and three subtypes of T2D were identified^1^: beta-cell dysfunction,^2^ insulin resistance combined with beta-cell dysfunction and^3^ insulin resistance. However, due to their unique population, the mean BMI of participants with T2D was 21±4.9 kg/m^2^. Our replication of their results in African immigrants with T2D who had a mean BMI of 29.8±4.5 kg/m^2^ represents a validation in Africans of the concept of three T2D subtypes.

A Ugandan study by Kibirige *et al* revealed a T2D subtype analogous to ID-T2D.^15^ Five hundred Ugandans with T2D confirmed by OGTT were divided into two groups: lean (BMI <25 kg/m^2^) and non-lean (BMI ≥25 kg/m^2^). One-third of the Ugandans were lean, and their beta-cell function was lower than in their non-lean counterparts. In the Africans in America cohort, all participants with both BMI <25 kg/m^2^ and T2D were in the ID-T2D subtype.

The IR-T2D subtype has also been described in South Africans and Ghanaians.^9 13^ In both South Africans and our participants with IR-T2D, the hyperinsulinemia–insulin resistance combination was associated with high BMI, central obesity and high VAT. In a study of Ghanaians enrolled in Research in Obesity and Diabetes among African Migrants (RODAM), impaired fasting glucose (IFG) was more highly correlated with insulin resistance than decreased beta-cell function.^9^ In the Africans in American study, 92% of participants with IR-T2D had IFG while only 40% of participants with ID-T2D had IFG.

Cluster analyses to identify diabetes subtypes have been conducted with South Asian, RODAM and Swedish data.^31 46 47^ These cluster analyses used homeostatic model assessment measurements of beta-cell function and insulin resistance as well as age, BMI, WC, HbA1c, fasting glucose, TG, HDL-C. In addition, through the use of GAD65 antibodies or C-peptide levels, all three cluster analyses identified individuals with type 1 diabetes or latent autoimmune diabetes (LADA). For two reasons, we believe no Africans in America participants had either type 1 diabetes or LADA. First, C-peptide levels were >600 pmol/L in the 40 participants with T2D who had C-peptide levels available. Second, both anti-insulin antibodies and GAD65 were negative in the eight participants who had diabetes and antibodies measured.

Cluster analyses conducted in India were the most similar to our results.^31^ They identified four clusters: cluster 1—severe insulin deficient diabetes, cluster 2—insulin resistant obese diabetes, cluster 3—combined insulin resistant and deficient diabetes and cluster 4—mild age-related diabetes (MARD). Cluster 2 and cluster 3 are analogous to our IR-T2D and ID-IR-T2D subtypes. This similarity may be based on the fact that both groups are experiencing a rise in T2D from beta-cell dysfunction. Cluster 4 is not concordant with our findings. In African immigrants, age increased across glucose tolerance categories but did not differ by T2DM subtype.

The RODAM study identified five diabetes clusters.^47^ The most common cluster was labeled Obesity-Related and occurred in 77% Ghanaians with diabetes, whereas the insulin-deficient cluster occurred in just 5%. The ID-T2D subtype was the most common in the Africans in America cohort and occurred in 40% of participants with T2D. However, the RODAM investigators may have underdiagnosed T2D. If true, this explains the small size of their insulin-deficient cluster. In the RODAM study, T2D was diagnosed by either medical history or fasting glucose ≥126 mg/dL. As undiagnosed T2D is common in Africa, individuals with undiagnosed T2D were almost certainly present in the RODAM cohort. Furthermore, early T2D was missed because FPG ≥126 mg/dL is a late manifestation of T2D.^1 5 6^

In the Swedish study, cluster design was based on the experience of high-income countries in which the etiology of T2D is usually obesity-induced insulin resistance.^46^ The authors’ state: ‘Model variables were selected on the premise that patients develop diabetes when they can no longer increase their insulin secretion (whatever the reason) to meet the increased demands imposed by obesity and insulin resistance’. In the Swedish context, there was no T2D subtype, which encompassed healthy weight individuals and insulin deficiency. However, our IR-T2D and ID+IR-T2D subtypes were analogous to their insulin-resistant and obesity-related clusters.

Overall, cluster analyses conducted in Africa, India and Europe mirror to varying degrees, our experience with T2D subtypes in African immigrants.^31 46 47^

### Strengths and limitations

This is the largest OGTT study in African immigrants in the world. As we did not rely on FPG or HbA1c to diagnose T2D, we were able to identify African immigrants with early phase T2D.^2 3 6^ In our study, 66% of participants with T2D were identified by 2-hour glucose alone and would have been missed if only FPG. Additionally, as multisampled OGTTs were performed with glucose and insulin levels obtained at each timepoint, insulin resistance and insulin deficiency, along with DI, could be determined.

However, as a cross-sectional study, we could not confirm conversion of NGT to pre-diabetes and to T2D. Nonetheless, prospective studies have demonstrated that a subset of individuals with NGT do transition to pre-diabetes and then to T2D.^41^,^44^

Another challenge was the use of a convenience sample. However, the Africans in American cohort appear representative of the African immigrant community in the USA. Consistent with census data, the majority of the cohort were male, came to the US since 2000, were from West and East Africa, and had a college education^48^ (online supplemental file 1). Furthermore, sickle cell and HbC trait prevalence were more common in immigrants from West and East Africa than Central Africa, and glucose-6-phosphate dehydrogenase deficiency was most common in participants from West Africa^49^ (online supplemental Table 4). In addition, the prevalence of T2D of 7% in the Africans in America cohort is similar to the prevalence of T2D in African immigrants living in Canada.^50^

## Conclusions

With the benefit of multisampled-OGTT, it was possible in African immigrants to characterize T2D from both physiologic and phenotypic perspectives as well as gain insight into modifiable factors.

Three types of T2D subtypes were identified: ID-T2D, ID+IR-T2D and IR-T2D. Insulin deficiency was the most prominent physiologic characteristic, and it occurred in two T2D subtypes, ID-T2D and ID+IR-T2D. As the insulin-deficiency T2D subtypes occurred across the BMI spectrum from healthy weight to obesity, consideration should be given, independent of BMI, to screening African immigrants for T2D. In addition, clinicians should understand that both of the T2D subtypes with insulin deficiency would benefit from early intervention with insulin therapy.

From a lifestyle perspective, weight gain, sedentary lifestyle and marriage all increased in prevalence across glucose tolerance categories from NGT to pre-diabetes to T2D. Therefore, African immigrants as soon as they enter the healthcare system should be counseled that life in America is associated with weight gain and decreased physical activity and therefore diabetogenic. Marriage should be seen as an opportunity. Family counseling could provide a potent educational opportunity. Combining family-based education with concepts learned from population-based physiologic studies, programs for improved T2D screening, treatment and even prevention can be instituted.

## Supplementary material

10.1136/bmjdrc-2025-005504 online supplemental file 1

## Data Availability

Data are available upon reasonable request.

## References

[1] (2025). IDF Diabetes Atlas - 11th Edition. https://diabetesatlas.org.

[2] Briker SM, Aduwo JY, Mugeni R (2019). A1C Underperforms as a Diagnostic Test in Africans Even in the Absence of Nutritional Deficiencies, Anemia and Hemoglobinopathies: Insight From the Africans in America Study. Front Endocrinol (Lausanne).

[3] Chivese T, Hirst J, Matizanadzo JT (2022). The diagnostic accuracy of HbA_1c_, compared to the oral glucose tolerance test, for screening for type 2 diabetes mellitus in Africa-A systematic review and meta-analysis. Diabet Med.

[4] Cowie CC, Rust KF, Byrd-Holt DD (2006). Prevalence of diabetes and impaired fasting glucose in adults in the U.S. population: National Health And Nutrition Examination Survey 1999-2002. Diabetes Care.

[5] Sumner AE, Thoreson CK, O’Connor MY (2015). Detection of abnormal glucose tolerance in Africans is improved by combining A1C with fasting glucose: the Africans in America Study. Diabetes Care.

[6] Kengne AP, Erasmus RT, Levitt NS (2017). Alternative indices of glucose homeostasis as biochemical diagnostic tests for abnormal glucose tolerance in an African setting. Prim Care Diabetes.

[7] Byiringiro S, Koirala B, Ajibewa T (2022). Migration-Related Weight Changes among African Immigrants in the United States. Int J Environ Res Public Health.

[8] ElSayed NA, McCoy RG, Aleppo G (2025). 2. Diagnosis and Classification of Diabetes: Standards of Care in Diabetes—2025. Diabetes Care.

[9] Meeks KAC, Stronks K, Adeyemo A (2017). Peripheral insulin resistance rather than beta cell dysfunction accounts for geographical differences in impaired fasting blood glucose among sub-Saharan African individuals: findings from the RODAM study. Diabetologia.

[10] Mukaz DK, Melby MK, Papas MA (2020). Diabetes and acculturation in African immigrants to the United States: analysis of the 2010-2017 National Health Interview Survey (NHIS). Ethn Health.

[11] Sewali B, Harcourt N, Everson-Rose SA (2015). Prevalence of cardiovascular risk factors across six African Immigrant Groups in Minnesota. BMC Public Health.

[12] Adeyemo AA, Zaghloul NA, Chen G (2019). ZRANB3 is an African-specific type 2 diabetes locus associated with beta-cell mass and insulin response. Nat Commun.

[13] Goedecke JH, Mendham AE (2022). Pathophysiology of type 2 diabetes in sub-Saharan Africans. Diabetologia.

[14] Joffe B (1992). Pathogenesis of non-insulin-dependent diabetes mellitus in the black population of southern Africa. The Lancet.

[15] Kibirige D, Sekitoleko I, Lumu W (2022). Understanding the pathogenesis of lean non-autoimmune diabetes in an African population with newly diagnosed diabetes. Diabetologia.

[16] PrayGod G, Filteau S, Range N (2021). β‐cell dysfunction and insulin resistance in relation to pre‐diabetes and diabetes among adults in north‐western Tanzania: a cross‐sectional study. *Tropical Med Int Health*.

[17] Ishimwe MCS, Wentzel A, Shoup EM (2021). Beta-cell failure rather than insulin resistance is the major cause of abnormal glucose tolerance in Africans: insight from the Africans in America study. *BMJ Open Diab Res Care*.

[18] Jagannathan R, DuBose CW, Mabundo LS (2020). The OGTT is highly reproducible in Africans for the diagnosis of diabetes: Implications for treatment and protocol design. Diabetes Res Clin Pract.

[19] Bigirimana SP, Ntabadde K, Smith GG (2025). Call for modification of the intermediate hyperglycemic classification of the 1 h-oral glucose tolerance test: insight from the Africans in America cohort Commentary. Diabetes Res Clin Pract.

[20] Hurston JS, Worthy CC, Huefner EA (2024). An Overview of Body Size Preference, Perception and Dissatisfaction in Sub-Saharan Africans Living in the United States. *Diabetes Metab Syndr Obes*.

[21] Kabeza CB, Ntabadde K, DuBose CW (2024). Determining the 1-hour post-load glucose which identifies diabetes in Africans: Insight from the Africans in America study. Diabetes Res Clin Pract.

[22] Hobabagabo AF, Osei-Tutu NH, Hormenu T (2020). Improved Detection of Abnormal Glucose Tolerance in Africans: The Value of Combining Hemoglobin A(1c) With Glycated Albumin. Diabetes Care.

[23] Shoup EM, Hormenu T, Osei-Tutu NH (2020). Africans Who Arrive in the United States before 20 Years of Age Maintain Both Cardiometabolic Health and Cultural Identity: Insight from the Africans in America Study. IJERPH.

[24] (1998). Executive Summary of the Clinical Guidelines on the Identification, Evaluation, and Treatment of Overweight and Obesity in Adults. Arch Intern Med.

[25] Kabakambira JD, Baker RL, Briker SM (2018). Do current guidelines for waist circumference apply to black Africans? Prediction of insulin resistance by waist circumference among Africans living in America. BMJ Glob Health.

[26] Alberti KGMM, Eckel RH, Grundy SM (2009). Harmonizing the metabolic syndrome: a joint interim statement of the International Diabetes Federation Task Force on Epidemiology and Prevention; National Heart, Lung, and Blood Institute; American Heart Association; World Heart Federation; International Atherosclerosis Society; and International Association for the Study of Obesity. Circulation.

[27] O’Connor MY, Thoreson CK, Ricks M (2014). Worse cardiometabolic health in African immigrant men than African American men: reconsideration of the healthy immigrant effect. Metab Syndr Relat Disord.

[28] Matsuda M, DeFronzo RA (1999). Insulin sensitivity indices obtained from oral glucose tolerance testing: comparison with the euglycemic insulin clamp. Diabetes Care.

[29] Utzschneider KM, Prigeon RL, Faulenbach MV (2009). Oral disposition index predicts the development of future diabetes above and beyond fasting and 2-h glucose levels. Diabetes Care.

[30] Harris PA, Taylor R, Thielke R (2009). Research electronic data capture (REDCap)--a metadata-driven methodology and workflow process for providing translational research informatics support. J Biomed Inform.

[31] Anjana RM, Baskar V, Nair ATN (2020). Novel subgroups of type 2 diabetes and their association with microvascular outcomes in an Asian Indian population: a data-driven cluster analysis: the INSPIRED study. *BMJ Open Diab Res Care*.

[32] Kagisaki T, Baden MY, Oyama A (2025). Impact of Weight Change on Type 2 Diabetes Risk: A Prospective Study in a Japanese Population. J Clin Endocrinol Metab.

[33] Bird SR, Hawley JA (2017). Update on the effects of physical activity on insulin sensitivity in humans. BMJ Open Sport Exerc Med.

[34] Duhuze Karera MG, Wentzel A, Ishimwe MCS (2023). A Scoping Review of Trials Designed to Achieve Remission of Type 2 Diabetes with Lifestyle Intervention Alone: Implications for Sub-Saharan Africa. Diabetes Metab Syndr Obes.

[35] Agyapong NAF, Annan RA, Apprey C (2020). Body Weight, Obesity Perception, and Actions to Achieve Desired Weight among Rural and Urban Ghanaian Adults. J Obes.

[36] Appiah CA, Otoo GE, Steiner-Asiedu M (2016). Preferred body size in urban Ghanaian women: implication on the overweight/obesity problem. Pan Afr Med J.

[37] Benkeser RM, Biritwum R, Hill AG (2012). Prevalence of overweight and obesity and perception of healthy and desirable body size in urban, Ghanaian women. Ghana Med J.

[38] Siervo M, Grey P, Nyan OA (2006). A pilot study on body image, attractiveness and body size in Gambians living in an urban community. Eat Weight Disord.

[39] Cerf ME (2013). Beta cell dysfunction and insulin resistance. Front Endocrinol (Lausanne).

[40] Esser N, Utzschneider KM, Kahn SE (2020). Early beta cell dysfunction vs insulin hypersecretion as the primary event in the pathogenesis of dysglycaemia. Diabetologia.

[41] Lyssenko V, Almgren P, Anevski D (2005). Predictors of and longitudinal changes in insulin sensitivity and secretion preceding onset of type 2 diabetes. Diabetes.

[42] Mason CC, Hanson RL, Knowler WC (2007). Progression to type 2 diabetes characterized by moderate then rapid glucose increases. Diabetes.

[43] Ohn JH, Kwak SH, Cho YM (2016). 10-year trajectory of β-cell function and insulin sensitivity in the development of type 2 diabetes: a community-based prospective cohort study. *The Lancet Diabetes & Endocrinology*.

[44] Tabák AG, Jokela M, Akbaraly TN (2009). Trajectories of glycaemia, insulin sensitivity, and insulin secretion before diagnosis of type 2 diabetes: an analysis from the Whitehall II study. Lancet.

[45] Agyemang C, Meeks K, Beune E (2016). Obesity and type 2 diabetes in sub-Saharan Africans - Is the burden in today’s Africa similar to African migrants in Europe? The RODAM study. BMC Med.

[46] Ahlqvist E, Storm P, Käräjämäki A (2018). Novel subgroups of adult-onset diabetes and their association with outcomes: a data-driven cluster analysis of six variables. *The Lancet Diabetes & Endocrinology*.

[47] Danquah I, Mank I, Hampe CS (2023). Subgroups of adult-onset diabetes: a data-driven cluster analysis in a Ghanaian population. Sci Rep.

[48] Tamir C (2022). Over half of black immigrants arrived in U.S. after 2000: pew research center. https://www.pewresearch.org/2022/01/20/over-half-of-black-immigrants-arrived-in-u-s-after-2000/.

[49] Piel FB, Patil AP, Howes RE (2013). Global epidemiology of sickle haemoglobin in neonates: a contemporary geostatistical model-based map and population estimates. Lancet.

[50] Creatore MI, Moineddin R, Booth G (2010). Age- and sex-related prevalence of diabetes mellitus among immigrants to Ontario, Canada. CMAJ.

