# Supplementary material for: African immigrants with type 2 diabetes present with three physiologic subtypes: implications for screening, diagnosis and treatment
Source: BMJ Open Diabetes Res Care. 2026 Mar 18;14(2):e005504. doi: 10.1136/bmjdrc-2025-005504 (PMC13007123; doi:10.1136/bmjdrc-2025-005504)

Supplement Table 1: Participant Characteristics

Parameter	Cohort n=633 100%	NGT n=386 61%	Prediabetes n=203 32%	Diabetes n=44 7%	*P*-value for trend
**Sex, Age and Body Size**					
**Male (%)**	62%	59%	65%	75%	0.025
**Age (y)**	39±11	36±10	43±11	45±10	<0.001
**BMI (kg/m^2)**	27.8±4.6	27.1±4.5	28.8±4.4	29.8±4.6	<0.001
**Obese (%) BMI≥30**	27%	23%	30%	41%	0.007
**WC (cm)**	91±12	88±12	94±11	99±10	<0.001
**VAT (cm^2) (n=555)**	97±67	77±55	122±73	158±64	<0.001
**Immigration and Sociodemographic Factors**					
**Immigration Age[2] (y)**	31±9	29±8	32±9	31±10	0.005
**Years in US[2]**	11±10	9±9	12±10	15±11	<0.001
**Weight gain in US (kg)2**	9±11	8±10	10±11	12±12	0.003
**Percent wgt gain (kg)2**	14±18	12±17	16±17	18±24	0.010
**Sedentary (%) (n=426)**	23%	18%	30%	31%	0.004
**Married (%)**	51%	44%	61%	68%	<0.001
**Alcohol Intake≥7 drk/wk**	6%	4%	6%	14%	0.057
**Smoker (%)**	4%	5%	2%	14%	0.779
**Income (>45K) (%)**	53%	52%	55%	59%	0.285
**College Graduate (%)**	73%	74%	72%	68%	0.387

[1]Chi-square and 1-Way ANOVA for categorical and continuous variables, respectively, **P*≤0.05. ***P*≤0.01, ****P*≤0.001.

(a for difference between NGT & prediabetes, b for difference between NGT & Diabetes, c for difference between Prediabetes & diabetes)

[2]Came to the US as adult≥18y

Supplement Table 2: Body and Physiologic Parameters by Glucose Tolerance Zone

	ID-NGT (n=166)	ID-PreD (n=78)_	ID-T2D (n=20)	ID+IR-T2D (n=10)	IR-T2D (n=13)	IR-PreD (n=73)	IR-NGT (n=57)	P-value	P-value
**Zone**	1			2	3				
	→→→→→→→→→→→→→→→→→→				←←←←←←←←←←←←←←←←←←←←				
**Parameter**	**NGT: BCI Range**	**PreD: BCI range**	**DM: BCI**	**DMI: IR+BCI**	**DM: IR**	**PreD: IR**	**NGT: IR**	**Trend 1 to 3**	**Trend 7 to 5**
**ISI**	0.31±0.08	0.30±0.08	0.25±0.11	0.26±0.13	0.53±0.09	0.87±0.35	1.23±0.48	0.132	<0.001
**IR (mat-inv)**	0.12±0.05	0.19±0.07	0.24±0.08	0.56±0.13	0.65±0.15	0.54±0.19	0.55±0.23	<0.001	0.078
**DI**	2.66±9.82	1.68±0.47	1.10±0.47	0.46±0.25	0.87±0.26	1.63±0.45	2.32±0.73	<0.001	<0.001
**BMI kg/m^2)**	26.1±4.4	27.0±3.5	26.8±2.9	32.2±3.9	32.3±4.6	30.6±4.3	29.6±4.7	0.011	0.063
**WC (cm)**	86±11	89±9	92±7	106±9	105±8	100±10	96±12	<0.001	0.004
**VAT (cm^2)**	62±48	100±63	132±60	151±46	203±65	151±84	121±60	<0.001	0.003

Supplement Table 3: Multivariate Logistic Regression to identify determinants of T2D

Variable	Odds Ratio	95% CI	P-value
**Model 1-Entire Cohort (n=633)**			
**Age**	1.05	1.02, 1.08	0.001
**Married**	1.74	0.87, 3.48	0.121
**Education***	1.39	0.71, 2.75	0.338
**Income**	1.04	0.54, 2.01	0.911
**Model 2-Cohort with Physical Activity Data (n= 426)**			
**Age**	1.04	1.01, 1.08	0.019
**Married**	1.85	0.79, 4.36	0.158
**Education***	1.53	0.68, 3.41	0.300
**Sedentary**	1.24	0.54, 2.84	0.612
**Income**	1.14	0.52, 2.51	0.750
**Model 3-Adult Immigrant participants (n= 464)**			
**Age**	1.05	1.01, 1.08	0.007
**Weight gain**	1.03	1.00, 1.06	0.048
**Married**	2.04	0.94, 4.44	0.074
**Education***	1.65	0.80, 3.41	0.180
**Income**	0.93	0.45, 1.92	0.838

*Not a college graduate

Supplement Table 4: Participant Characteristics by African Region of Origin

Characteristics[1]	Total n=633 100%	West n=310 49%	East[2] n=214 34%	Central n=109 17%	*P*-value [3,4]
**Male (%)**	62%	63%	58%	70%	0.094
**Age (y)**	39±11	40±11	38±10	39±12	0.354
**BMI (kg/m^2)**	27.9±4.6	28.4±4.7	26.8±3.9	28.7±5.1	<0.001a***, c*
**Hematologic/Genetic Factors**					
**Sickle Cell or HbC Trait**	16%	21%	8%	19%	<0.001
**G6PD Deficiency (n=267)[5]**	10%	16%	5%	7%	0.010
**Sociodemographic Factors**					
**Arrived US in 2000 or later**	68%	63%	74%	69%	0.037
**Adult Immigrant (%)**	83%	79%	86%	88%	0.042
**College Graduate (%)**	73%	76%	71%	68%	0.182

[1]Data presented as mean±SD or Percent

[2]The 10 individuals from Southern African countries were analyzed with the East African group.

[3]One-way ANOVA or chi-square as appropriate

[4]One-way ANOVA notation: a: difference between West & East Africa, b: difference between West & Central Africa, c: difference between East & Central Africa *<0.05,**<0.01, ***<0.001

[5]Assay done in 267 consecutively enrolled participants

Supplement Figure 1

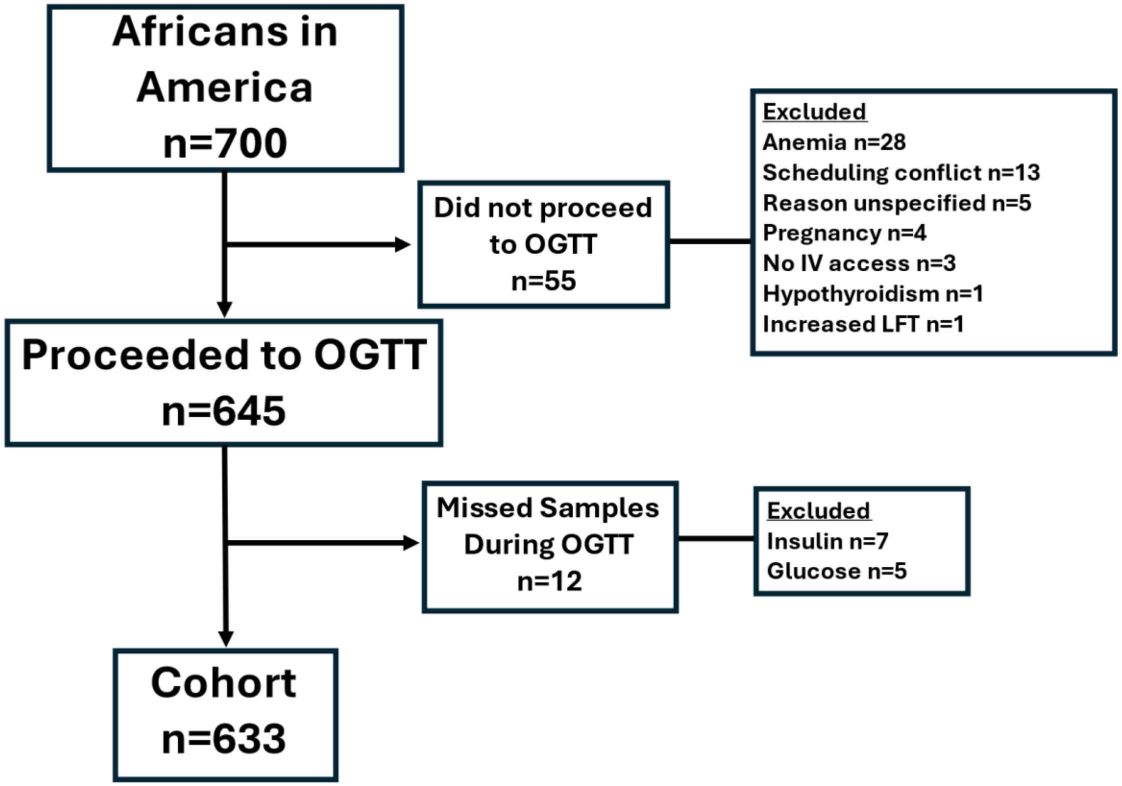

Supplement Figure 2

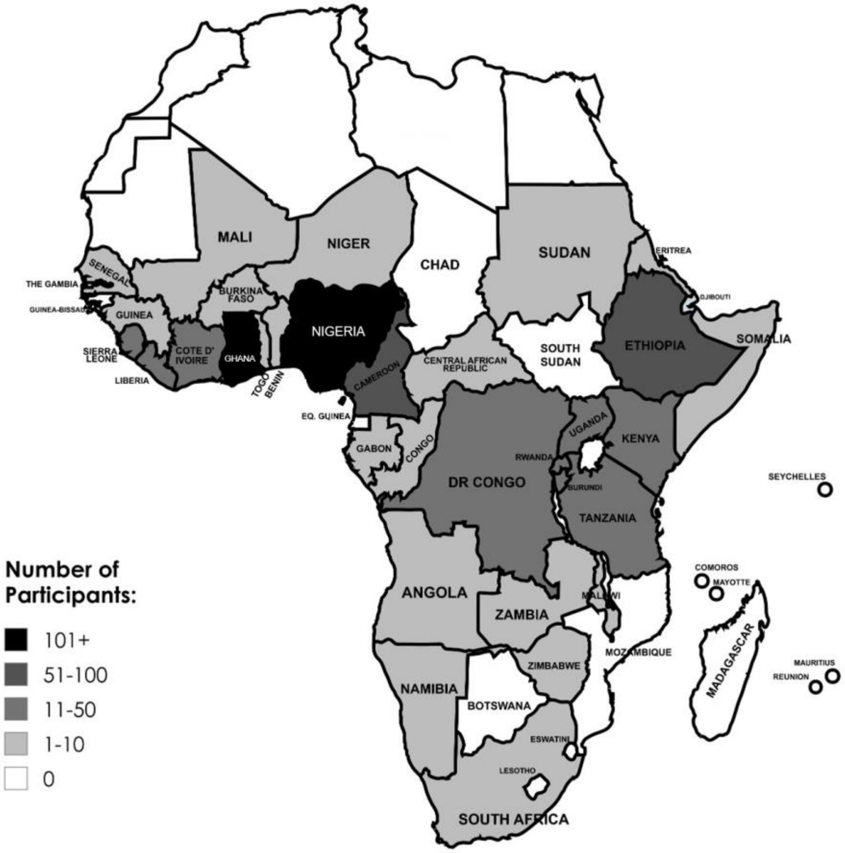

Supplement: online supplemental file 1 [file bmjdrc-14-2-s001.pdf]
